# 82-kDa choline acetyltransferase and SATB1 localize to β-amyloid induced matrix attachment regions

**DOI:** 10.1038/srep23914

**Published:** 2016-04-07

**Authors:** Warren Winick-Ng, Fabiana A. Caetano, Jennifer Winick-Ng, Trevor M. Morey, Bryan Heit, R. Jane Rylett

**Affiliations:** 1 Department of Physiology and Pharmacology, Schulich School of Medicine & Dentistry, University of Western Ontario, London, Ontario, N6A 5C1 Canada; 2 Molecular Medicine Group, Robarts Research Institute, University of Western Ontario, London, Ontario, N6A 5C1 Canada; 3 London Health Sciences Centre, London, Ontario, N6A 5W9 Canada; 4 Department of Microbiology and Immunology, Schulich School of Medicine & Dentistry, University of Western Ontario, London, Ontario, N6A 5C1 Canada

## Abstract

The M-transcript of human choline acetyltransferase (ChAT) produces an 82-kDa protein (82-kDa ChAT) that concentrates in nuclei of cholinergic neurons. We assessed the effects of acute exposure to oligomeric amyloid-β_1–42_ (Aβ_1–42_) on 82-kDa ChAT disposition in SH-SY5Y neural cells, finding that acute exposure to Aβ_1–42_ results in increased association of 82-kDa ChAT with chromatin and formation of 82-kDa ChAT aggregates in nuclei. When measured by chromatin immunoprecipitation with next-generation sequencing (ChIP-seq), we identified that Aβ_1–42_ -exposure increases 82-kDa ChAT association with gene promoters and introns. The Aβ_1–42_ -induced 82-kDa ChAT aggregates co-localize with special AT-rich binding protein 1 (SATB1), which anchors DNA to scaffolding/matrix attachment regions (S/MARs). SATB1 had a similar genomic association as 82-kDa ChAT, with both proteins associating with synapse and cell stress genes. After Aβ_1–42_ -exposure, both SATB1 and 82-kDa ChAT are enriched at the same S/MAR on the APP gene, with 82-kDa ChAT expression attenuating an increase in an isoform-specific APP mRNA transcript. Finally, 82-kDa ChAT and SATB1 have patterned genomic association at regions enriched with S/MAR binding motifs. These results demonstrate that 82-kDa ChAT and SATB1 play critical roles in the response of neural cells to acute Aβ -exposure.

Cholinergic neurons are critical for communicating information to a wide range of target cells using the neurotransmitter acetylcholine (ACh)^1^,^2^. In brain, cholinergic neurons regulate functions such as cognition, motor control and sleep^3^,^4^. Neurological disorders such as mild cognitive impairment (MCI) and Alzheimer’s disease (AD) are characterized by changes in the function of cholinergic neurons; cholinergic neuron activity may be enhanced initially due to synaptic plasticity, followed by loss of function of these neurons related at least in part to the toxic effects of β-amyloid peptides (Aβ) that are produced by cleavage of amyloid precursor protein (APP)^5^,^6^,^7^. Oligomeric Aβ can cause cellular toxicity, oxidative stress and aberrant signal transduction leading to inhibition of synaptic plasticity and neuron degeneration^7^,^8^,^9^,^10^,^11^. Cholinergic neurons in the basal forebrain are particularly sensitive to Aβ, resulting in reductions in choline uptake, choline acetyltransferase (ChAT) activity and ACh release^12^,^13^.

ChAT, which catalyzes the production of ACh from choline and acetyl Coenzyme A, is transcribed from the cholinergic gene locus and encoded by at least 8 transcripts that are generated by alternative splicing or differential utilization of several non-coding exons located in the ChAT gene promoter^14^,^15^. All ChAT transcripts encode a 69-kDa protein, but in humans the M-type transcript has an additional in-frame translation initiation site that can also result in the production of an 82-kDa ChAT protein^14^,^15^. The 82-kDa ChAT protein is identical to 69-kDa ChAT except that it bears an 118 amino acid residue amino-terminal extension. While both 69-kDa ChAT and 82-kDa ChAT are transported into the nucleus of cells, 69-kDa ChAT is a nucleocytoplasmic shuttling protein that moves out of the nucleus and is concentrated in the cytoplasm^16^. By comparison, 82-kDa ChAT is localized predominantly in the nucleus facilitated by it having two strong nuclear localization signal (NLS) motifs^16^,^17^. Our finding that ChAT is in nuclei of cholinergic neurons is the first report of a transmitter-synthesizing enzyme in nuclei of neurons, but reports of nuclear localization of other transmitter enzymes are emerging; glutaminase, which synthesizes the excitatory amino acid transmitter glutamate, is also in both nuclei and cytoplasm of neurons^18^. Little is known about the functional roles for these enzymes in nuclei, but it is likely not related to neurochemical transmission. There are also no data about regulatory events that control their relative distribution between cytoplasm and nucleus.

Importantly, in cholinergic neurons in necropsy human brain, the nuclear localization of 82-kDa ChAT is reduced with increasing age and in patients with MCI or early AD^19^. Recent studies highlight that the expression of several genes related to APP processing^20^, including the cholinergic-specific protein the sodium-coupled choline transporter CHT^21^, are altered in cells expressing 82-kDa ChAT. A critical observation made in our laboratory is that when 82-kDa ChAT is expressed in neurons cultured from AD-model mice that express mutant human APP and Presenilin 1 (PS1) transgenes, β-secretase activity is reduced leading to decreased Aβ production^20^. The mechanisms by which 82-kDa ChAT promotes these changes in gene transcription, and how this is impacted by the subcellular redistribution of the enzyme seen in aging and MCI are unknown. Therefore, the current studies were designed to assess nuclear distribution of 82-kDa ChAT and if this is altered by cellular perturbations, such as acute exposure of neural cells to oligomeric Aβ_1–42_.

The findings in this manuscript demonstrate that 82-kDa ChAT aggregates in nuclei of neural cells and associates with chromatin at gene promoters and exons after human neuroblastoma SH-SY5Y cells are exposed to oligomeric Aβ_1–42_. We also show that 82-kDa ChAT co-localizes with special AT-rich binding protein 1 (SATB1), an organizing component of the nuclear matrix. SATB1 anchors chromatin loops to the nuclear matrix as part of scaffolding/matrix attachment regions (S/MARs), and can recruit histone deacetylases (HDACs) or histone acetyltransferases (HATs) specifically to these regions^22^,^23^,^24^. S/MARs are anchored to the nuclear matrix by SATB1 binding to local and distant AT-rich DNA sequences, forming chromatin loops that are accessible for chromatin modifying factors to repress or activate transcription^22^,^24^. SATB1 had a similar genomic distribution as 82-kDa ChAT and both proteins were associated with an *in silico* predicted S/MAR on the *APP* gene, leading to changes in gene expression after exposure to Aβ_1–42_. Finally, both SATB1 and 82-kDa ChAT had patterned genomic associations at predicted S/MARs. The data presented in this study establishes that acute exposure of neural cells to Aβ can induce an epigenetic response that is dependent on the expression of 82-kDa ChAT.

## Results

### 82-kDa ChAT sub-nuclear localization is altered after acute exposure of cells to Aβ

**Figure 1:**
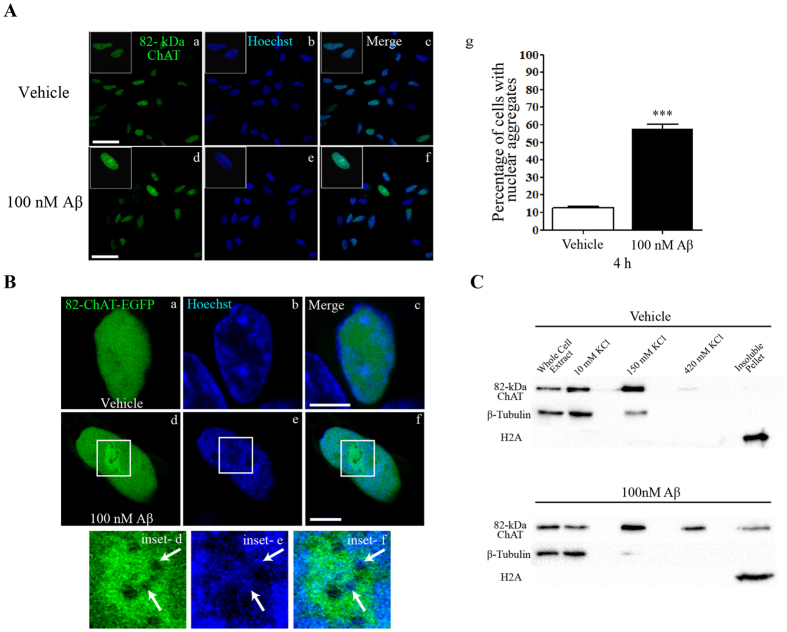
82-kDa ChAT forms nuclear aggregates after exposure to Aβ. (**A**) SH-SY5Y cells stably expressing 82-kDa ChAT were exposed to either vehicle (F12 media; a–c) or 100 nM oligomeric Aβ_1–42_ for 4 h (d–f). Aβ-treated cells had nuclear aggregates of 82-kDa ChAT protein. The left panel shows 82-kDa ChAT staining, center panel shows staining with Hoechst dye to label DNA, and right panel is the overlay; n = 5, with at least 6 cells imaged per treatment; scale bar 20 μm. (g) Quantification of the number of cells with nuclear aggregates of 82-kDa ChAT, n = 8 with at least 100 cells counted per treatment. ***p < 0.001 (Student’s *t*-test). (**B**) SH-SY5Y cells were transiently transfected with 82-kDa ChAT-eGFP and treated with either vehicle (a–c) or 100 nM oligomeric Aβ_1–42_ (d–f) for 4 h, then live cells were exposure to Hoechst dye for 20 min prior to imaging. Aβ-exposed cells had aggregates of 82-kDa ChAT-eGFP (inset on d) surrounding areas with little ChAT-eGFP and aggregates of Hoechst dye (inset on e,f). Scale bar 5 μm; n = 5. (**C**) Immunoblot showing cell fractionation of SH-SY5Y cells stably expressing 82-kDa ChAT treated with either vehicle or 100 nM oligomeric Aβ_1–42_ for 4 h. Aβ-treated cells showed an increase in 82-kDa ChAT protein levels present in the 420 mM KCl fraction, with a small amount of protein detected in the insoluble protein fraction. β-tubulin and histone H2A were used as controls for the 10 mM KCl and insoluble protein fractions, respectively, n = 8.

To evaluate the subcellular localization of 82-kDa ChAT and changes that may occur after acute exposure of cells to oligomeric Aβ_1–42_, we imaged retinoic acid-differentiated SH-SY5Y cells stably expressing heterologous 82-kDa ChAT at 4 h after exposure to either vehicle or Aβ_1–42_ by confocal microscopy. We observed diffuse, slightly punctate staining for 82-kDa ChAT in the nuclei of vehicle-treated cells, with a small amount of cytoplasmic staining (Fig. 1A[a–c]). After exposure of cells to 100 nM oligomeric Aβ_1–42_ for 4 h, immunofluorescence staining of cells for 82-kDa ChAT reveals highly-fluorescent aggregates of the protein within the nuclei of many cells (Fig. 1A[d–f]). We determined that 58 ± 2% of Aβ-treated cells had at least 1 nuclear aggregate of 82-kDa ChAT, compared to 13 ± 1% of vehicle-treated cells (Fig. 1A[g]).

Next, we determined whether these Aβ-induced aggregates are also formed in live cells that express 82-kDa ChAT as a fusion protein with enhanced green fluorescent protein (82-kDa ChAT-eGFP; Fig. 1B). Consistent with findings in the previous experiment, vehicle-treated cells had a diffuse distribution of 82-kDa ChAT-eGFP in nucleus (**a–c**), whereas cells treated for 4 h with 100 nM Aβ_1–42_ had nuclear aggregates of 82-kDa ChAT-eGFP (Fig. 1B[d–f]). As a DNA marker, we treated live cells with 625 ng/mL Hoechst 33342 for 20 min prior to imaging. When the Aβ-induced aggregates of 82-kDa ChAT-eGFP were imaged at higher magnification, it is apparent that there were areas within the aggregates that have lower levels of 82-kDa ChAT staining (Fig. 1B[inset d]) and that these were enriched with Hoechst dye (Fig. 1B[inset e–f]).

With the Aβ-induced changes in 82-kDa ChAT sub-nuclear localization, as well as the presence of DNA within the aggregates, we asked whether 82-kDa ChAT could be associated with chromatin. We used cell fractionation analysis to identify subcellular compartments where 82-kDa ChAT was localized (Fig. 1C). In agreement with the confocal images, a large proportion of the 82-kDa ChAT protein is found in the fraction containing soluble nuclear proteins (150 mM KCl). In control cells, a small immuno-positive band for 82-kDa ChAT protein is found in the euchromatic chromatin (420 mM KCl) fraction, but no immuno-positive band is seen in the insoluble pellet fraction containing heterochromatic associated, nuclear matrix associated and insoluble proteins. After 4 h exposure of cells to 100 nM Aβ_1–42_, there was a strong 82-kDa ChAT protein immuno-positive band in the 420 mM KCl fraction and a small band detected in the insoluble pellet fraction. As a control, β-tubulin was found mainly in the 10 mM KCl (cytosolic) fraction, while histone H2A was found only in the insoluble pellet fraction.

### 82-kDa ChAT increases its association with gene introns and promoters after Aβ -exposure

**Figure 2:**
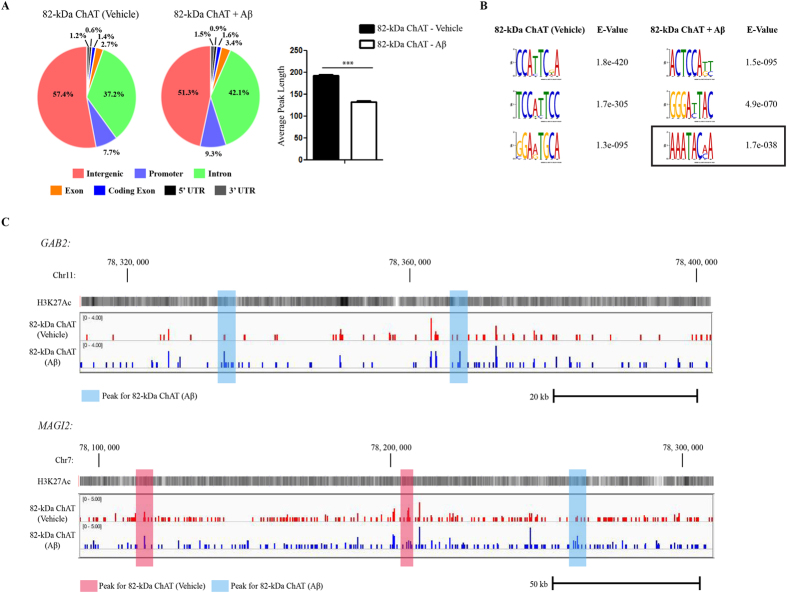
82-kDa ChAT has altered genome association after exposure of cells to Aβ. (**A**) ChIP-seq was performed for SH-SY5Y cells stably expressing 82-kDa ChAT treated with either vehicle or 100 nM oligomeric Aβ_1–42_ for 4 h. Genomic features identified for 82-kDa ChAT. Both treatment groups had over 50% of the peaks at intergenic regions, but Aβ_1–42_ treatment increased association with introns, promoters and exons. Average peak length for 82-kDa ChAT after either vehicle or Aβ_1–42_ -exposure was also tested. Treatment with Aβ_1–42_ significantly decreased the 82-kDa ChAT peak length. ***p < 0.001 (Student’s *t*-test). (**B**) Top DREME motif hits for 82-kDa ChAT after vehicle or Aβ_1–42_ -exposure of cells. The top motifs for both treatments revealed a TC_2-3_AT motif, while Aβ_1–42_ -treatment also revealed a known SATB1 binding motif (boxed region). (**C**) Example ChIP-seq tracks for 82-kDa ChAT after vehicle or Aβ_1–42_ -exposure of cells for regions of *GAB2* and *MAGI2* genes. Peaks are highlighted in blue for Aβ_1–42_ peaks and red for vehicle peaks. H3K27Ac is overlaid to show active transcription initiation sites.

We found that 82-kDa ChAT is associated with chromatin and has several putative DNA binding motifs in its N-terminal extension as predicted by the *in silico* tools BindN+^25^ and DNABindR^26^ (Fig. S1). These include tandem (S/T)PXX and XPRK motifs which bind AT-rich DNA at the minor groove^27^,^28^, and a basic residue region with high DNA binding prediction. In addition, the crystal structure of human 69-kDa ChAT shows that it also has surface-accessible regions of basic residues that could facilitate this association with DNA^29^. Therefore we performed chromatin immunoprecipitation followed by next-generation sequencing (ChIP-seq) for 82-kDa ChAT in cells treated for 4 h with either vehicle or 100 nM Aβ_1–42_ (Fig. 2). We found 7460 peaks for 82-kDa ChAT after vehicle treatment, and 6345 peaks after Aβ_1–42_ treatment, with 250 peaks overlapped and an additional 414 peaks within 200 nt of one another. We observed that 57.4% of peaks were found in intergenic regions after vehicle-treatment, compared to 51.3% after Aβ_1–42_ -exposure (Fig. 2A). The decrease in intergenic regions after exposure to Aβ_1–42_ was explained by an increase in the percentage of the peaks found in introns (42.1%) compared to vehicle treatment (37.2%). In addition, the percentage of peaks found in promoters and exons was also increased after Aβ_1–42_ -exposure (9.3% and 3.4%, respectively) compared to vehicle treatment (7.7% and 2.7%, respectively). We also observed that the average peak length was significantly reduced after Aβ_1–42_ treatment for 82-kDa ChAT (132.6 ± 2.2 nucleotides (nt)) compared to vehicle treatment (192.6 ± 3.2 nt). Smaller peak sizes suggest targeted genomic associations with sequence-specific DNA targets^30^.

Next, we used the discriminative DNA motif discovery (DREME) tool^31^ for motif discovery (Fig. 2B) and observed that the top DREME motifs contained the motif TC_2-3_AT in both conditions. Additionally, we identified a known binding motif ((A/T)_3-6_C(A/T)_3-n_) for the chromatin organizing protein SATB1^22^, as one of the top motifs after exposure of cells to Aβ_1–42_. We analyzed data sets obtained from gene expression microarray experiments carried out in our laboratory comparing IMR32 human neuroblastoma cells either expressing or not expressing 82-kDa ChAT^20^ (NCBI GEO database accession number: GSE3506) and identified that the expression of *SATB1* was increased 5.5-fold in cells stably expressing 82-kDa ChAT. We confirmed that SATB1 mRNA expression was increased in SH-SY5Y cells stably expressing 82-kDa ChAT (1.47 ± 0.15-fold, p < 0.05, n = 3). Examples of ChIP-seq tracks from random genomic targets for 82-kDa ChAT, treated with vehicle or Aβ_1–42_, are shown in Fig. 2C for *GAB2* and *MAGI2* genes, with H3K27 acetylation (H3K27ac) overlaid to show active transcription initiation sites. Additional tracks can be found in Fig. S2.

### Aβ-induced 82-kDa ChAT aggregates co-localize with special AT-rich binding protein 1

**Figure 3:**
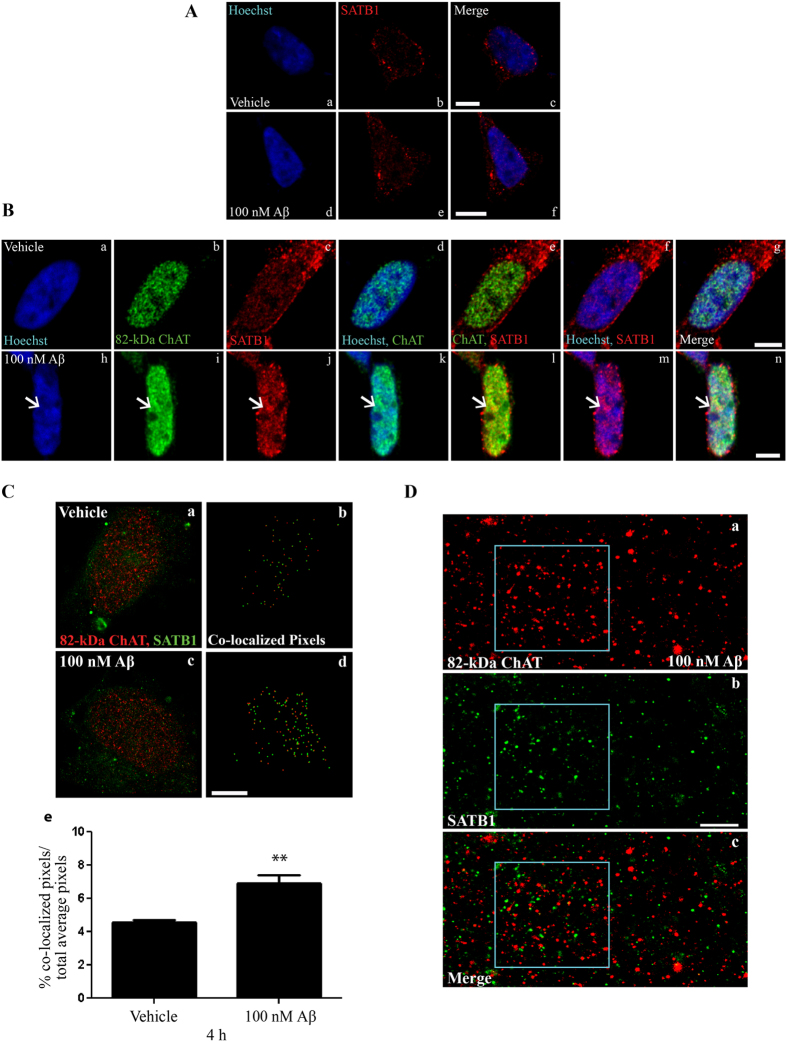
Aβ-induced 82-kDa ChAT aggregates are co-localized with SATB1. (**A**) SH-SY5Y cells expressing empty vector were treated with either vehicle (a–c) or 100 nM oligomeric Aβ_1–42_ (d–f) for 4 h. SATB1 was localized to nucleus and cytoplasm, with diffuse expression in nucleus in both vehicle and Aβ-treated cells. The left panel shows Hoechst staining, center panel shows SATB1 immunostaining, and right panel is the overlay. (**B**) SH-SY5Y cells stably expressing 82-kDa ChAT treated with vehicle (a–g) or 100 nM oligomeric Aβ_1–42_ (h–n) for 4 h. After treatment with Aβ, 82-kDa ChAT (i) showed nuclear aggregates (arrows). SATB1 nuclear expression was greater than in control cells and showed aggregate formation (j) in the same region as the 82-kDa ChAT aggregates (l). Scale bar 5 μm; n = 5, with at least 6 cells imaged per treatment. (**C**) Co-localization analysis for SR-GSDIM images of 82-kDa ChAT and SATB1. (b) and (d) are the co-localization results for the respective SR-GSDIM image of vehicle (a) and 100 nM Aβ_1–42_ (c) treatments. (e) The percentage of co-localized pixels was increased significantly in Aβ-treated cells. **p < 0.01 (Student’s *t*-test, n = 5). (**D**) Digitally magnified SR-GSDIM images for 82-kDa ChAT (a) and SATB1 (b) levels after 100 nM oligomeric Aβ_1–42_ for 4 h. The boxed region shows an Aβ-induced aggregate of 82-kDa ChAT and SATB1. The overlay (c) revealed SATB1 protein within the 82-kDa ChAT accumulation. Scale bar 500 nm; n = 6, with at least 4 cells imaged per treatment.

We identified by ChIP-seq that 82-kDa ChAT associated with motifs that had sequence similarity to SATB1 binding motifs, and that *SATB1* expression was increased after 82-kDa ChAT was stably expressed in SH-SY5Y cells. Thus, we targeted SATB1 and S/MARs as potentially having a role in Aβ-induced 82-kDa ChAT aggregation. First, the expression of 82-kDa ChAT in cells influences the subcellular localization of SATB1 protein in response to acute Aβ treatment (Fig. 3). When assessed by confocal microscopy, SATB1 protein is distributed in cells between the cytoplasm and nucleus, with the protein level in the nucleus being higher at the nuclear periphery. This subcellular distribution of SATB1 did not differ between vehicle-treated cells that do not express 82-kDa ChAT (Fig. 3A[a–c]) and cells that do express the ChAT protein (Fig. 3B[c]), and is not altered by Aβ_1–42_ treatment for vector-expressing cells (Fig. 3A[d–f]). Importantly however, in Aβ-treated cells that express 82-kDa ChAT, the SATB1 protein in the nucleus forms aggregates (Fig. 3B[j]). As seen previously, 82-kDa ChAT protein distribution is diffuse and slightly punctate in nuclei of vehicle-treated cells (Fig. 3B[a–g]), but in cells treated with Aβ_1–42_ aggregates of 82-kDa ChAT (Fig. 3B[h–n]) and SATB1 co-localize in the nucleus (Fig. 3B[l]).

To further evaluate the interaction between 82-kDa ChAT and SATB1 after cellular exposure to Aβ_1–42_, we used super-resolution ground-state depletion microscopy followed by individual molecule return (SR-GSDIM) (Fig. 3C). First, we analysed the SR-GSDIM images for co-localization of 82-kDa ChAT and SATB1, indicated by the pixels in Fig. 3C[b,d] and defined as a distance between the two proteins of less than 2/3^rd^ protein size (see Methods for more detail). In control cells, there is a small amount of co-localization of the two proteins (Fig. 3C[a,b]) that is increased significantly after exposure to Aβ_1–42_ (Fig. 3C[c,d]). Quantification reveals 4.6 ± 0.14% of co-localized pixels in control images, with this increased significantly by 50% (6.9 ± 0.51%) in Aβ-treated cells (Fig. 3C[e]). While this method allowed co-localization analysis between the two proteins, it was difficult to observe the nuclear aggregates under these conditions. Therefore, we digitally magnified the SR-GSDIM images to a 500 nm scale, in order to evaluate the relationship between the nuclear aggregates of 82-kDa ChAT protein (Fig. 3D[a]) and SATB1 protein (Fig. 3D[b]). When SATB1 aggregate localization was overlaid with that of 82-kDa ChAT, there appeared to be a close association between the two proteins, compared to regions outside of the aggregate (Fig. 3D[c]).

To better understand aspects of the underlying mechanisms, we determined if modulators of the NAD-dependent deacetylase sirtuin 1 (SIRT1) would alter 82-kDa ChAT and SATB1 localization. SIRT1 can deacetylate histones and other proteins, and has been shown previously to deacetylate SATB1 leading to the formation of S/MARs^32^. We used two pharmacological approaches to accomplish this, by adding either the SIRT1 activator resveratrol^33^ or the specific inhibitor 6-chloro-2,3,4,9-tetrahydro-1H-carbazole-1-carboxamide (EX527)^34^.

**Figure 4:**
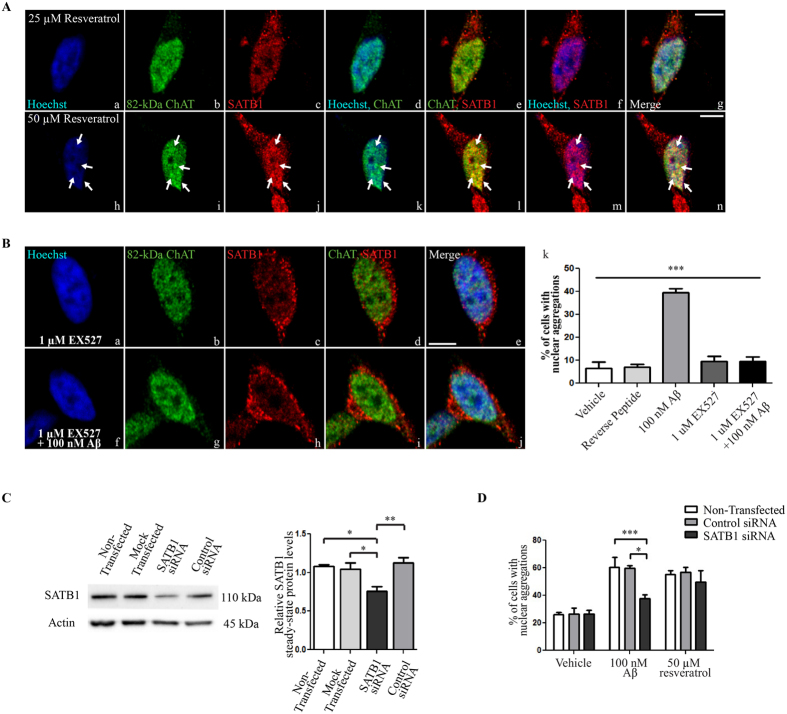
Activation of SIRT1 is required for 82-kDa ChAT/SATB1 aggregate formation. (**A**) SH-SY5Y cells stably expressing 82-kDa ChAT and treated with the SIRT1 activator resveratrol at 25 μM for 5 h (a–g) did not result in formation of aggregates of either 82-kDa ChAT or SATB1 proteins. Treatment with 50 μM resveratrol promoted aggregate formation (arrows on h–n) of both 82-kDa ChAT (i) and SATB1 (j) proteins that were co-localized (l,n). Scale bar 5 μm; n = 5 with at least 5 cells imaged for each treatment. (**B**) Cells were pre-treated with the SIRT1 inhibitor EX527 for 1 h prior to 4 h treatment with vehicle (a–e) or 100 nM oligomeric Aβ_1–42_ (f–j). Pre-treatment of cells with EX527 prior to Aβ-exposure prevented the formation of 82-kDa ChAT aggregates (k). n = 5, ***p < 0.001 (one-way ANOVA). (**C**) Representative western immunoblot showing SATB1 steady-state protein levels in SH-SY5Y cells stably expressing 82-kDa ChAT and either non-transfected, mock transfected with no siRNA, transfected with untargeted control siRNA, or transfected with siRNA targeted to SATB1 for 24 h. Quantification revealed a 29% reduction in SATB1 protein expression compared to controls. n = 5, **p < 0.01, *p < 0.05 (one-way ANOVA with Tukey’s *post hoc* test). (**D**) SH-SY5Y cells stably expressing 82-kDa ChAT were either non-transfected, transfected with untargeted control siRNA, or transfected with siRNA targeted to SATB1 for 24 h, followed by either 100 nM oligomeric Aβ_1–42_ for 4 h or 50 μM resveratrol for 5 h. The number of cells positive for nuclear aggregates of 82-kDa ChAT were quantified by a blinded observer as a percentage of the total number of cells counted. After Aβ_1–42_ -exposure, the percentage of 82-kDa ChAT aggregates were significantly reduced after SATB1 siRNA knockdown compared to non-transfected or control siRNA transfected cells. We observed no significant differences for resveratrol or vehicle treatment. n = 3 independent experiments with at least 100 cells counted per treatment group, ***p < 0.001, *p < 0.05 (two-way ANOVA with Bonferroni’s *post hoc* test).

We treated cells with resveratrol to activate SIRT1 in the absence of Aβ_1–42_ (Fig. 4A). We chose doses of resveratrol that were shown previously to have effects on SIRT1 activity in SH-SY5Y cells^35^,^36^. No aggregates of either of the two proteins are observed in the nuclei of cells treated for 5 h with 25 μM resveratrol (Fig. 4A[a–g]). However, when the concentration of resveratrol is increased to 50 μM, numerous aggregates of both 82-kDa ChAT and SATB1 are observed in cell nuclei (Fig. 4A[h–n]).

In other experiments, we pre-treated cells with the SIRT1-specific inhibitor EX527 for 1 h prior to exposure to Aβ_1–42_ (Fig. 4B). Importantly, aggregates of 82-kDa ChAT are not observed in cells that were pre-treated with 1 μM EX527 for 1 h prior to the addition of either vehicle (Fig. 4B[a–e]) or 100 nM Aβ_1–42_ for 4 h (Fig. 4B[f–j]). We found that 10 ± 2% of cells contain aggregates after pre-treatment with EX527 alone. Compared to Aβ_1–42_ alone (40 ± 2%), the number of aggregates is significantly reduced in cells pre-treated with EX527 prior to exposure to Aβ_1–42_ (10% ± 2%) (Fig. 4B[k]). As an additional control we included treatment of cells with a reverse Aβ_1–42_ peptide, which did not result in the production of significant 82-kDa ChAT aggregates compared to control.

Finally, we assessed whether SATB1 was necessary for the formation of the Aβ-induced aggregates of 82-kDa ChAT using small-interfering RNA (siRNA) targeted to SATB1 (Fig. 4C). Compared to non-transfected (1.08 ± 0.03), mock transfected (1.05 ± 0.08), and non-targeted control siRNA (1.13 ± 0.07), we observed a 29% reduction in SATB1 protein levels after transfection of cells with SATB1 siRNA for 24 h (0.76 ± 0.06). This resulted in a significant reduction in SATB1 steady-state protein levels compared to untransfected, mock transfected and control siRNA cells. We next counted the percentage of cells that had at least one nuclear aggregate in cells that were treated with either vehicle for 4 h, 100 nM Aβ_1–42_ for 4 h or 50 μM resveratrol for 5 h (Fig. 4D). We found no significant differences for non-transfected cells (26 ± 2%), cells transfected with control siRNA (26 ± 4%), or transfection with SATB1 siRNA (26 ± 3%) in vehicle-treated cells. For cells that were treated with Aβ_1–42_, transfection with SATB1 siRNA significantly reduced the number of 82-kDa ChAT aggregates observed to (37 ± 3%) compared to cells that were non-transfected (60 ± 7%) or cells transfected with control siRNA (60 ± 2%). Surprisingly, after resveratrol treatment we observed no significant differences for SATB1 siRNA transfected cells (50 ± 8%) compared to control siRNA (57 ± 4%) or non-transfected (55% ± 3%) cells. These data indicate that SATB1 is required for the Aβ_1–42_ -induced aggregates. In addition, SIRT1 activation can produce aggregates of 82-kDa ChAT/SATB1, but this is not dependent on SATB1 expression.

### 82-kDa ChAT and SATB1 associate with chromatin at synapse and cell stress genes

**Figure 5:**
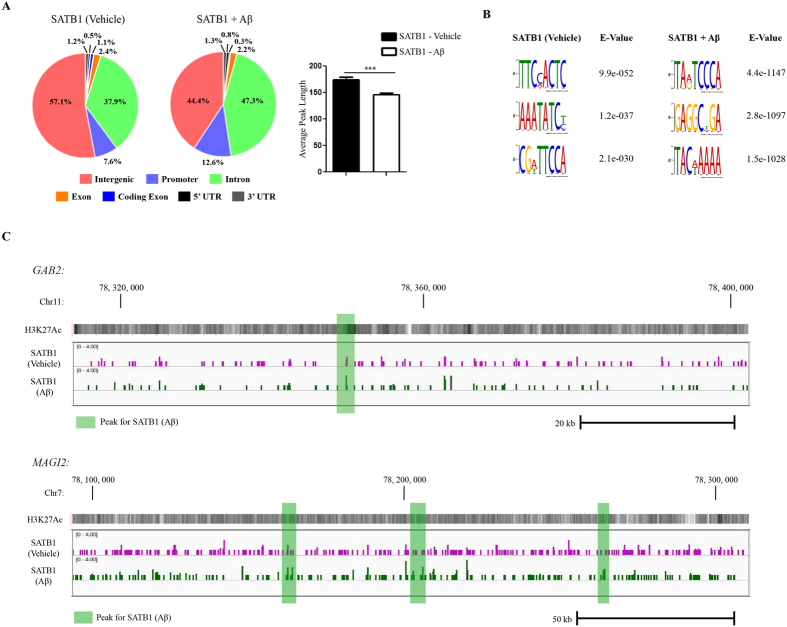
SATB1 has altered genome association after exposure of cells to Aβ. (**A**) ChIP-seq analysis was performed for SATB1 using SH-SY5Y cells stably expressing 82-kDa ChAT and exposed to either vehicle or 100 nM oligomeric Aβ_1–42_. Genomic features identified for SATB1. Aβ treatment increases SATB1 association with introns and promoters. Treatment with Aβ_1–42_, significantly decreased average peak length for SATB1. ***p < 0.001 (Student’s *t*-test). (**B**) The top DREME motif hits for SATB1 after vehicle or Aβ_1–42_ -exposure revealed a TC_2-3_AT motif, and a known SATB1 binding motif. (**C**) Example ChIP-seq tracks for SATB1 after vehicle or Aβ_1–42_ -exposure of cells for regions of *GAB2* and *MAGI2* genes corresponding to the same regions in Fig. 2C. Peaks are highlighted in green for Aβ_1–42_ peaks. There were no vehicle related peaks in these regions (see Fig. S2 for examples). H3K27Ac is overlaid to show active transcription initiation sites.

82-kDa ChAT and SATB1 proteins are co-localized after cells are exposed to Aβ, therefore we performed ChIP-seq for SATB1 as a comparison to 82-kDa ChAT (Fig. 5). We found 2884 peaks for SATB1 after vehicle treatment, and 5857 peaks for SATB1 after Aβ_1–42_ treatment, with 36 peaks overlapped and 35 additional peaks within 200 nt of one another (Fig. 5A). Similar to 82-kDa ChAT, we observed that 57.1% of peaks were found in intergenic regions after vehicle treatment for SATB1, compared to 44.4% after Aβ_1–42_ -exposure. Moreover, the decrease in intergenic regions after exposure of cells to Aβ_1–42_ was explained by an increase in peaks found in introns (47.3%) and promoters (12.6%) compared to vehicle (37.9% and 7.6%, respectively). We again observed that the average peak length was significantly reduced after Aβ_1–42_ treatment for SATB1 (146.6 ± 2.2 nt) compared to vehicle treatment (173.9 ± 4.9 nt) (Fig. 5A). Using the DREME tool, we observed that both vehicle and Aβ_1–42_ treatment of cells resulted in the same TC_2-3_AT motif seen in the 82-kDa ChAT samples (Fig. 5B). In addition, peaks for SATB1 from cells treated with either vehicle or Aβ_1–42_ contained the SATB1 motif (A/T)_3-n_C(A/T)_3-6_ seen in the Aβ-exposed 82-kDa ChAT sample. Examples of ChIP-seq tracks from random genomic targets for SATB1 at *GAB2* and *MAGI2* genes and treated with vehicle or Aβ are shown in Fig. 5C. Additional tracks can be found in Fig. S2.

**Figure 6:**
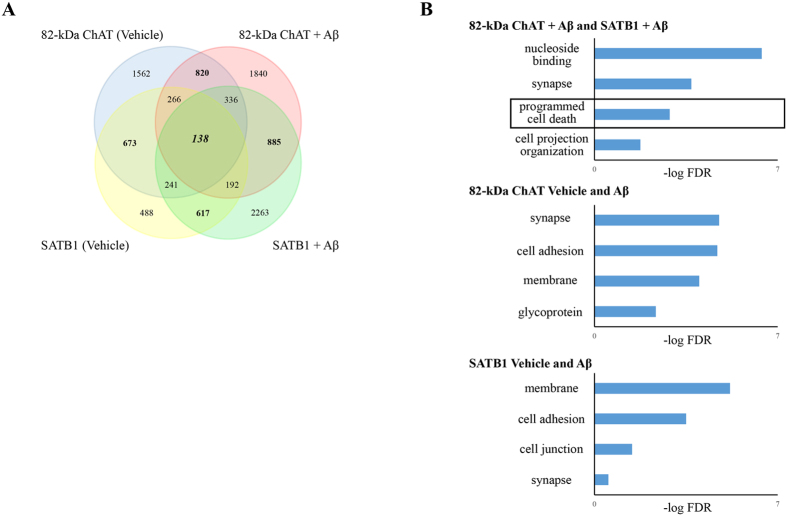
82-kDa ChAT and SATB1 associate with shared cell membrane and stress related genes after Aβ-exposure. (**A**) Number of genes associated with ChIP-seq peaks for 82-kDa ChAT and/or SATB1 after treatment of cells with vehicle or 100 nM oligomeric Aβ_1–42_. There was increased gene association between the proteins after exposure of cells to Aβ. (**B**) Gene ontology analysis for groups of gene associations found by ChIP-seq. 82-kDa ChAT and SATB1 had significant associations with genes encoding proteins involved in synapse function, cell adhesion and cell membrane function with both treatments. For genes associated with both 82-kDa ChAT and SATB1 after exposure to Aβ_1–42_, there were significant gene associations for nucleoside binding and programmed cell death (box). Gene ontology terms are presented with the -log value of the false discovery rate (FDR).

We annotated peaks contained in intergenic components for both proteins, and found that, in agreement with the genomic features, there was a higher number of associated genes after exposure of cells to Aβ_1–42_ for both 82-kDa ChAT (4507) and SATB1 (4672) compared to vehicle treatment (3700 and 2615, respectively) (Fig. 6A). An important finding was that there were more genes in common between 82-kDa ChAT and SATB1 after exposure of cells to Aβ_1–42_ (885) than there were for common genes for either 82-kDa ChAT (820) or SATB1 (617) in either of the conditions. We found 138 genes associated with all treatments. We next evaluated what functional groups the genes were associated with using the Database for Annotation, Visualization and Integrated Discovery (DAVID) server^37^,^38^ (Fig. 6B). After exposure of cells to Aβ_1–42_, we found significant gene ontology (GO) terms related to nucleoside binding, synapse, and cell projection for both 82-kDa ChAT and SATB1. Similarly, we found synapse, membrane and cell adhesion GO terms for SATB1 and 82-kDa ChAT regardless of treatment with either vehicle or Aβ_1–42_. We also found the GO term ‘regulation of programmed cell death’ (GO: 0043067) for both 82-kDa ChAT and SATB1 after Aβ-exposure. In addition to these GO terms we also identified several genes previously identified by a meta-analysis of genome wide association studies assessing AD risk^39^, including *BIN1, CR1, EFNA5, GAB2, MAGI2, MTHFD1L* and *PRUNE2*. We also found several APP binding and metabolism related genes in several conditions (Table 1). It is of interest that *ADAM10, ADAM17*, *APBA2, APPBP2*, and *RTN1* were previously identified as upregulated by 82-kDa ChAT in a gene expression microarray^20^. We also identified several additional APP related genes, such as *ADAM12*, *APBB1*, *APBB2* and *NAE1*. Finally, we identified *APP* itself, which had peaks for both 82-kDa ChAT and SATB1 only after exposure of cells to Aβ_1–42_.

### 82-kDa ChAT and SATB1 are associated with *APP* and alter gene expression

**Figure 7:**
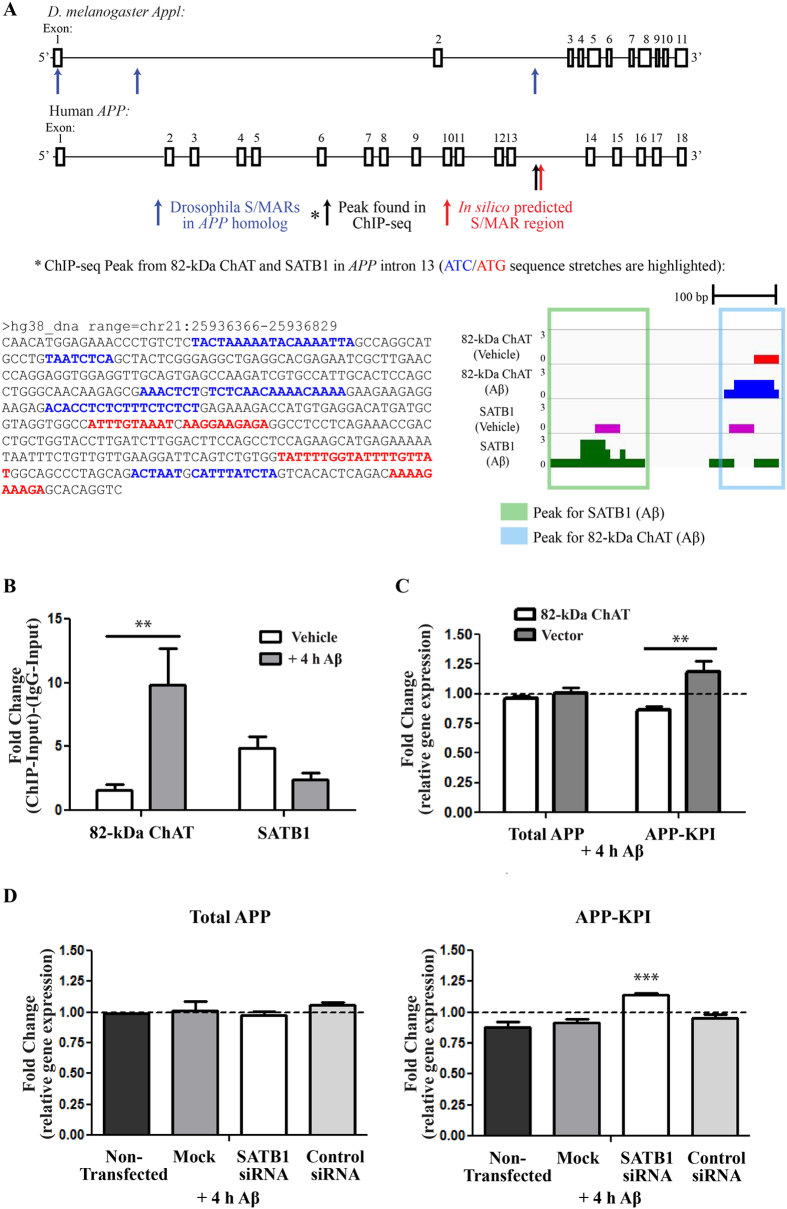
82-kDa ChAT and SATB1 associate with the *APP* gene after Aβ-exposure and alter gene expression. (**A**) Schematic showing exons (boxes) and introns (horizontal lines) for the *D. melanogaster Appl* and human *APP* genes. Blue arrows indicate S/MARs regions on *Appl* identified by Pathak *et al*.^37^, the red arrow a predicted S/MAR in *APP* by *in silico* analysis, and the black arrow a ChIP-seq peak found for 82-kDa ChAT and SATB1 after Aβ_1–42_ -exposure. ATC/ATG rich sequences are highlighted in this region. ChIP-seq tracks for 82-kDa ChAT and SATB1 are also shown for this region. (**B**) Verification of 82-kDa ChAT and SATB1 ChIP-seq peaks on *APP*. 82-kDa ChAT showed significantly increased fold-enrichment after exposure of cells to 100 nM oligomeric Aβ_1–42_ for 4 h, while SATB1 did not significantly change its association with the region after either vehicle or Aβ_1–42_ treatment. n = 6, **p < 0.01 (two-way ANOVA with Bonferroni’s *post-hoc* test). (**C**) *APP* steady-state mRNA expression in 82-kDa ChAT or vector-expressing cells after exposure to 100 nM oligomeric Aβ_1–42_. Data are presented as fold-enrichment compared to reference (GAPDH) mRNA levels and vehicle-treated cells (dashed-line). Vector-expressing cells showed higher steady-state mRNA levels of isoforms containing KPI compared to 82-kDa ChAT-expressing cells. n = 4, **p < 0.01 (two-way ANOVA with Bonferroni’s *post-hoc* test). (**D**) *APP* steady-state mRNA expression in 82-kDa ChAT-expressing cells after exposure to 100 nM oligomeric Aβ_1–42_ after either no transfection, a mock transfection, transfection with untargeted control siRNA, or transfection with siRNA targeted to SATB1. Data are presented as fold-enrichment compared to reference (GAPDH) mRNA levels and vehicle-treated cells (dashed-line). There were no significant changes in total *APP* mRNA levels for any of the treatments. Cells transfected with SATB1 siRNA showed higher steady-state mRNA levels of APP isoforms containing KPI compared to non-transfected 82-kDa ChAT-expressing cells. n = 5, ***p < 0.001 (one-way ANOVA with Dunnett’s *post-hoc* test).

To validate peaks found within the ChIP-seq dataset, we chose to examine a peak found after exposure of cells to Aβ_1–42_ for both SATB1 and 82-kDa ChAT on the *APP* gene at intron 13 (Table 1), that were within 160 nt of one another. *APP* has previously been found to have altered transcription in AD patients following transcriptome analysis^40^, and after MAPK activation by anisomycin in SH-SY5Y cells^41^. Combined with recent evidence showing 82-kDa ChAT is implicated in alterations in APP processing^20^, we identified *APP* as a potential target for an 82-kDa ChAT/SATB1 S/MAR. Using the *in silico* MAR-Wiz S/MAR prediction tool^42^, we identified a predicted S/MAR ~55 kb upstream of the 3′ end of *APP* that was ~2 kb upstream of the ChIP-seq peaks (Fig. 7A). Pathak *et al*.^43^ identified DNA associated with nuclear matrix S/MARs from *D. melanogaster* embryos using next generation sequencing (NCBI Sequence Read Archive accession number: SRX443533). We identified 3 peaks in the *APP* homolog *App1* associated with potential S/MARs, with one peak found in the same region as the ChIP-seq peaks (Fig. 7A). In addition, the region containing the ChIP-seq peaks had several sequence similarities with known SATB1 binding motifs^22^. We tested for enrichment of both SATB1 and 82-kDa ChAT using ChIP followed by quantitative PCR (ChIP-qPCR) (Fig. 7B). For 82-kDa ChAT, we observed a 1.6 ± 0.5-fold enrichment after vehicle treatment, which was significantly higher after Aβ_1–42_ -exposure (9.8 ± 2.9-fold). We found a 4.9 ± 0.9-fold enrichment for SATB1 after vehicle treatment, compared to 2.4 ± 0.5-fold after exposure of cells to Aβ_1–42_, though there was no statistical difference between the treatments.

Next, we used RT-qPCR to assess any potential changes to *APP* steady-state mRNA levels (Fig. 7C). Using SH-SY5Y cells stably expressing either 82-kDa ChAT or empty vector, we found no significant changes in total *APP* steady-state mRNA levels after exposure for 4 h 100 nM Aβ (0.96 ± 0.02-fold, 1.00 ± 0.05-fold respectively). Several groups have reported that *APP* mRNA isoforms (including APP751 and 770) containing the Kunitz-type serine protease inhibitor domain (APP-KPI) are increased in AD patients, which correlates to increases in Aβ_1–42_^44^,^45^. Therefore we tested whether there were any Aβ-induced changes in APP-KPI mRNA steady-state levels, and observed that there is significantly increased expression of APP-KPI mRNA steady-state levels in vector-expressing cells (1.18 ± 0.09-fold) compared to cells expressing 82-kDa ChAT (0.86 ± 0.03-fold).

Finally, we assessed whether siRNA knockdown targeted to SATB1 could alter steady-state levels of total *APP* mRNA or the Aβ-induced change in APP-KPI mRNA observed in 82-kDa ChAT-expressing cells (Fig. 7D). After exposure for 4 h with 100 nM Aβ_1–42_, we observed no significant changes in total *APP* steady-state mRNA levels for SH-SY5Y cells stably expressing 82-kDa ChAT after a mock transfection, control siRNA or siRNA targeted to SATB1 compared to cells that were not transfected. When we measured APP-KPI steady-state mRNA levels, we observed significantly increased expression of APP-KPI levels in cells expressing 82-kDa ChAT and transfected with SATB1-targeted siRNA (1.14 ± 0.01-fold) compared to non-transfected cells (0.88 ± 0.05-fold). There were no significant differences for mock transfected cells (0.91 ± 0.03-fold) or control siRNA transfected cells (0.95 ± 0.03-fold) compared to non-transfected cells.

### 82-kDa ChAT and SATB1 associate with chromatin at S/MARs

Because SATB1 is involved in the anchoring of S/MARs to the nuclear matrix, and we show that both SATB1 and 82-kDa ChAT associated with a predicted S/MAR on *APP*, we asked whether 82-kDa ChAT may also be localized at S/MARs. We used motifs used by the *in silico* MAR-Wiz tool^42^, along with known SATB1 motifs^22^ and select motifs discovered in the *D. melanogaster* S/MAR dataset^43^ (Table S3). In addition to the analysis of 82-kDa ChAT and SATB1, we also included a ChIP-seq dataset for Alpha Thalassemia/Mental Retardation Syndrome X-Linked (ATRX) (NCBI GEO database accession number: GSE22162)^46^, as this protein is involved in chromatin reorganization, but has not previously been associated with S/MARs^46^,^47^,^48^. ATRX had a total of 6368 peaks, with an average peak length of 483.4 ± 2.4nt. Due to the large differences in peak lengths, we examined the number of motifs found in each peak weighted by the inverse of the peak length divided by a scaling factor of 100 (Fig. S4). Overall, we found that ATRX (0.9 ± 0.002%) had significantly fewer weighted motifs/peak compared to both 82-kDa ChAT and SATB1 after both vehicle and Aβ_1–42_ -exposure. Surprisingly, SATB1 after Aβ_1–42_ treatment of cells (1.4 ± 0.007%) was also significantly reduced compared to all treatments other than ATRX. We also tested the weighted motifs/peak for the 5 motifs that had the highest number of associated peaks. For motifs 1 (an origin of replication motif) and 20–21 (SATB1 motifs), we saw a similar pattern as the overall data, indicating that 82-kDa ChAT and SATB1 peaks significantly associated with genomic regions containing S/MAR binding sites.

As a control, we also determined the number of motifs in each peak of the ATRX-binding G-quadruplex motif: G_3+_N_1–20_G_3+_N_1–20_G_3+_N_1–20_G_3+_^46^ (Table S5). For ATRX we found 732 peaks with at least one G-quadruplex motif and 1327 motifs total. By comparison, for 82-kDa ChAT we found only 113 peaks and 204 motifs after vehicle treatment, and 89 peaks with 121 motifs after Aβ_1–42_ -exposure. For SATB1, we found 60 peaks and 109 motifs after vehicle treatment, and 30 peaks with only 41 motifs after Aβ_1–42_ -exposure. These data indicate that G-quadruplex motifs are enriched in ATRX, but not for 82-kDa ChAT or SATB1.

**Figure 8:**
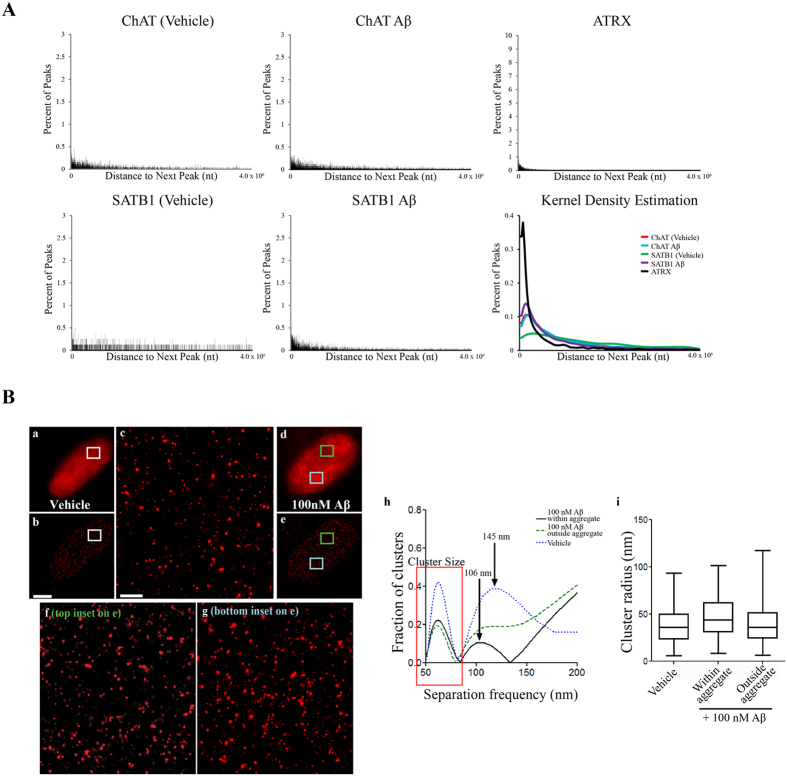
Analysis of the inter-peak spacing of 82-kDa ChAT and SATB1. (**A**) Distribution of inter-peak distances for ChIP-seq peaks. For each graph, the shape of the graph was estimated using a kernel density estimation. Indicated by a single curve, the kernel density estimation revealed regular patterned spacing for ATRX and 82-kDa ChAT, and for SATB1 after Aβ_1–42_ cell treatment. SATB1 did not show any patterned inter-peak spacing after vehicle treatment. (**B**) SR-GSDIM imaging for SH-SY5Y cells stably expressing 82-kDa ChAT treated with for 4 h either vehicle or 100 nM Aβ. Epi-fluorescence images show diffuse staining for vehicle-treated cells (a) and nuclear aggregates after Aβ treatment (d). SR-GSDIM images show diffuse punctate staining for both vehicle (b) and Aβ (e) treated cells. Enlarged ROIs for vehicle-treated cell (c), and a region either outside (f) or within (g) an Aβ-induced 82-kDa ChAT aggregate. Scale bars on (b) 3 μm and on (c) 500 nm; n = 6 with at least 4 cells per treatment. For each ROI, clusters were identified and the average inter-cluster distance was quantified (h). Vehicle-treated ROIs or Aβ-induced aggregates showed even spacing between clusters (arrows on h), while clusters from ROIs outside an aggregate did not show even spacing. Cluster size did not vary significantly for any of the conditions (red box on h,i). n = 3 with 2 cells used for each ROI in each condition.

Pathek *et al*.^43^ found that the inter-MAR distance in the *D. Melanogaster* genome ranged from <1 kb to 150 kb, with an average distance of 16 kb and a peak of inter-MAR distances at approximately 5 kb. Due to the potential association with S/MARs, we also assessed whether there was any pattern to the spacing between successive peaks across each chromosome (Fig. 8A). We found that the distance between peaks for each treatment ranged from <1 kb to 28 Mb, except for ATRX which had an upper range of 56 Mb. For 82-kDa ChAT, 75% of peaks were less than 1.4 Mb apart after either vehicle or Aβ_1–42_ treatment of cells. For SATB1, 75% of peaks were less than 1.1 Mb apart following Aβ_1–42_ -exposure, with this number increasing to 3.8 Mb after vehicle treatment. For ATRX, 75% of peaks were less than 0.5 Mb apart. Through kernel density estimation with an upper bound of 4.0 Mb, we found that there were peaks in the distributions of the distance between successive peaks, indicative that regular patterned spacing was present. For 82-kDa ChAT, the peak occurred at a spacing of 125.0 kb after vehicle treatment, and 123.9 kb after Aβ_1–42_ -exposure. We also found regular spacing for SATB1 after exposure to Aβ_1–42_ (92.5 kb) and for ATRX (31.2 kb). Interestingly, we did not observe any pattern to the inter-peak distances for SATB1 after vehicle exposure. These data show that, after Aβ exposure, both SATB1 and 82-kDa ChAT have regular patterned spacing on the genome similar to S/MAR genome spacing.

As a comparison to the genomic patterned spacing observed for 82-kDa ChAT, we tested whether there was patterning to the nuclear protein localization using SR-GSDIM. Images of 82-kDa ChAT immunostaining in nuclei of vehicle-treated cells by either epi-fluorescence or SR-GSDIM microscopy are shown in Fig. 8B[a,b respectively], with an enlarged region of interest (ROI, Fig. 8B[c]). For Aβ-treated cells, we observed nuclear aggregates of 82-kDa ChAT when cells were imaged in both epi-fluorescence mode (Fig. 8B[d]) and in SR-GSDIM [**e**] mode. SR-GSDIM imaging showed 82-kDa ChAT predominantly in multimeric clusters in vehicle-treated (Fig. 8B[c]) and Aβ-exposed regions not associated with an aggregate (Fig. 8B[f]), or within an aggregate (Fig. 8B[g]). Using a Fourier transform, we quantified the spacing between clusters in these ROIs. Two peaks were apparent in the Fourier analysis (Fig. 8B[h]), the first corresponding to the average cluster size and the second indicating regular spacing between clusters. We found that in 38% of vehicle-treated cells and in 10% of regions containing an Aβ-induced aggregate the clusters displayed even spacing, while regions in Aβ-exposed cells not associated with an aggregate did not contain any regular patterning. In vehicle-treated cells, the peak of inter-cluster distance was found to be 145 nm, while in regions containing an Aβ-induced aggregate this distance was reduced to 106 nm. We measured the size of the individual clusters and found no significant difference in any of the conditions (box on Fig. 8B[h,i]).

## Discussion

In the present study, we explored the novel finding that 82-kDa ChAT can facilitate and participate in an epigenetic response in Aβ-exposed human neural cells. Specifically, we found that 1) nuclear localized 82-kDa ChAT forms aggregates in Aβ_1–42_ -treated cells; 2) 82-kDa ChAT is associated with chromatin after exposure of cells to Aβ_1–42_; and 3) 82-kDa ChAT increases its association with gene introns and promoters after Aβ-exposure of cells. 82-kDa ChAT protein aggregates in neural cell nuclei are co-localized with SATB1, a protein involved in anchoring DNA to the nuclear matrix for either chromatin activation or repression. SATB1 had a similar gene association as 82-kDa ChAT with or without exposure to Aβ_1–42_. Both 82-kDa ChAT and SATB1 interact with chromatin at a predicted S/MAR in the *APP* gene, leading to altered gene expression of specific *APP* mRNA isoforms. Finally, 82-kDa ChAT and SATB1 are associated with chromatin at regions enriched with S/MAR motifs, and these regions have regular patterned expression on the genome.

Initial characterization of the effects of Aβ-treatment on 82-kDa ChAT localization showed protein aggregates in SH-SY5Y cell nuclei after only 4 h. Moreover, 82-kDa ChAT protein is associated with chromatin, and ChIP peaks associated with 82-kDa ChAT have a significant number of motifs with sequence similarity to a known SATB1 binding motif. SATB1 anchors chromatin to the nuclear matrix as part of S/MARs, a process involving targeted chromatin looping^22^,^23^,^24^. Chromatin is highly organized into transcriptionally repressive and active loops during interphase and in non-dividing cells^49^. Heterochromatic regions of S/MARs recruit either polycomb repressive complexes for histone H3K27 tri-methylation (H3K27me3) or K9-histone methyltransferase (HMT) for H3K9me3 resulting in transient or constitutive repression of transcription, respectively^22^,^23^,^50^. Conversely, activated S/MARs recruit the HAT p300/CBP, and K4-HMTs for H3K4me3, and RNA polymerase II (RNA Pol II) for transcriptional activation^49^,^50^.

There are several examples of changes in gene expression related to S/MAR formation during cell stress. SMAR1, which also binds nuclear matrix, recruits HDAC1 to repress the *BAX* and *PUMA* promoters during mild DNA damage, but is sequestered from these genes after extensive DNA damage, resulting in apoptosis^51^. In relation to APP processing, over-expressing SIRT1 in APP/PS1 transgenic mice leads to activation of the α-secretase gene *ADAM10* and a subsequent reduction in the amyloidogenic APP processing pathway^52^. Interestingly, we found that SATB1/82-kDa ChAT aggregates were formed after activation of SIRT1 independent of Aβ_1–42_ treatment, and SATB1 siRNA knockdown did not alter the SIRT1-dependent aggregate formation. Xue *et al*.^32^ showed that SIRT1 deacetylates SATB1 to promote the formation of S/MARs at the β-globulin locus, though SIRT1 has many other non-SATB1 targets that are likely contributing to the Aβ-induced SATB1/82-kDa ChAT aggregate formation seen in this study. We demonstrated previously that *ADAM10* transcription levels were elevated in IMR32 cells expressing 82-kDa ChAT, while β-secretase levels and activity were reduced in neurons cultured from APP/PS1 transgenic mouse brain that have been transduced to express 82-kDa ChAT^20^. In the present study, we found that *ADAM10* has ChIP-seq peaks for 82-kDa ChAT after vehicle and Aβ-exposure, along with several other genes encoding proteins that interact with APP. Further work is needed to determine if there may be 82-kDa ChAT and S/MAR involvement in these responses to cell stress.

The present study found that 82-kDa ChAT and SATB1 are both enriched at an *in silico* predicted S/MAR on the *APP* gene after exposure of cells to Aβ_1–42_. While *APP* mRNA expression is altered in AD patients^40^,^53^, and increased after MAPK activation by anisomycin in SH-SY5Y cells^41^, other studies suggest that there may be an increase in the isoforms that contain KPI in AD patients^44^,^45^. These studies show that total *APP* mRNA steady-state levels are unchanged, but this alternative expression pattern correlates to an increase in soluble Aβ_1–42_ and to severity of cognitive impairment^44^,^45^. We found previously that total *APP* mRNA steady-state levels were not changed when 82-kDa ChAT was expressed in primary neurons from APP/PS1 mice^20^. Our current findings complement these studies, as we demonstrate that cells expressing 82-kDa ChAT have lower steady-state levels of KPI-containing isoforms of *APP* compared to vector-expressing cells, with no change in total *APP* mRNA levels. The change in APP-KPI was prevented when we used siRNA targeted to SATB1, demonstrating that SATB1 is also necessary for this change in gene expression. We postulate that expression of 82-kDa ChAT protein may contribute to preventing the increased production of these KPI-containing isoforms in neurons after exposure to environmental stress, with this potentially disrupted when the subcellular localization of 82-kDa ChAT is altered in aging, MCI or AD^19^. It is important to note that the KPI-containing *APP* mRNA isoform is alternatively spliced to include exon 7^54^, while 82-kDa ChAT and SATB1 bind to a region in intron 13. While a linear genomic relationship between these two regions is unlikely, it will be important for future studies to understand how chromatin may be organized in these regions to elucidate how the interaction of SATB1 and 82-kDa ChAT may influence the steady-state mRNA levels of APP-KPI.

When we analyzed ChIP-seq datasets for 82-kDa ChAT and SATB1, we saw a number of similarities including increases in promoter and intragenic targets. We observed that both proteins had smaller peak sizes after exposure to Aβ_1–42_, which suggests targeted genomic associations as small peak sizes are associated with sequence-specific DNA targets^30^. We showed that 82-kDa ChAT and SATB1 have associations with sequences are enriched with S/MAR binding motifs, in particular SATB1 binding motifs and an origin of replication sequence. However, there were also important differences between the datasets. For 82-kDa ChAT there were a similar number of peaks after vehicle or Aβ-exposure, but for SATB1 there were more than double the number of associations found after Aβ-exposure. In addition, we showed that a subset of the genome associations for 82-kDa ChAT had regular patterned spacing and, at the nano-resolution level, 82-kDa ChAT forms patterned clusters with the distance between clusters decreasing after Aβ-exposure. We found a subset of SATB1 genome associations with regular patterned spacing after cells were exposed to Aβ_1–42_, but not for vehicle-treated cells. We observed that SIRT1 activation stimulates SATB1 and 82-kDa ChAT aggregate formation, and both SIRT1 activation and Aβ-exposure result in SATB1 entry into the nuclear interior. Taken together, these data suggest that SATB1 is activated to bind to chromatin at S/MARs after exposure to Aβ_1–42_, resulting in alternative utilization and movement of 82-kDa ChAT to these regions. Thus, we propose a model where 82-kDa ChAT is constitutively associated with chromatin, but is brought to SATB1 anchored S/MARs after Aβ-exposure.

The current study demonstrates that 82-kDa ChAT participates in, and is required for, the binding of S/MARs to the nuclear matrix in human neural cells after acute exposure to oligomeric Aβ_1–42_. Our data suggests that cholinergic neurons can have an epigenetic response to Aβ-exposure, and given that the nuclear levels of 82-kDa ChAT decline with increasing age and the onset of cognitive impairment^19^, the loss of this epigenetic response may have implications for the onset or progression of MCI and AD. Future work will be important for understanding the signalling pathways involved in the formation of these S/MARs after Aβ-exposure. It will also be important to assess the effective outcomes of S/MAR formation on cellular processes, especially for genes identified in this study related to AD risk and APP processing. These insights could lead to the identification of new biomarkers, as well as therapeutic targets for the etiology of AD and other cognitive disorders.

## Materials and Methods

### Cell culture and transfection

SH-SY5Y cells from American Type Culture Collection (Manassus, VA) were grown on 35 mm glass-bottom plates (0.14 mm thick; MatTek Corp., Ashland, MD, USA) for live cell imaging, or cells stably expressing heterologous 82-kDa ChAT^20^ were plated on coverslips for immunostaining. Cells were differentiated using 10 μM all-*trans*-retinoic acid for 3 days, which produces substantial morphological and biochemical cholinergic differentiation of cells^55^. For live imaging, cells were transfected with a plasmid encoding an 82-kDa ChAT-eGFP fusion protein or the peGFP vector (Clontech Laboratories Inc., Mountain View, CA, USA) using Lipofectamine 2000 (Invitrogen, Burlington, ON, Canada). Aβ oligomers were prepared as described^56^ from lyophilized Aβ_1–42_ or the reverse peptide purchased from rPeptide (Bogart, GA, USA). Cells were treated with either 100 nM oligomeric Aβ_1–42_ or F12 media (vehicle), or resveratrol (Sigma-Aldrich, Oakville, ON, Canada) with or without pre-treatment with EX527 (Sigma-Aldrich) for the indicated times and concentrations. Cells in some experiments were transfected with either scrambled control siRNA or specific siRNA duplexes targeted against human SATB1 (Santa-Cruz Biotechnology, Dallas, TX, USA) for 24 h with Lipofectamine RNAiMAX (Invitrogen) prior to treatment with Aβ or resveratrol. As an additional negative control, cells were mock transfected with Lipofectamine containing no siRNA.

### Antibodies

The 82-kDa ChAT protein was detected with a custom rabbit polyclonal antibody CTab^57^. Other antibodies used were: anti-SATB1 (sc-5990) and anti-β-actin (sc-1616-R) (Santa-Cruz Biotechnology), anti-β-tubulin (2146S; Cell Signaling, Danvers, MA, USA) and anti-histone H2A (H2A) (PA1–41004; Thermo Scientific). AlexaFluor 647 and AlexaFluor 542-conjugated secondary antibodies (Invitrogen) were used for immunostaining visualization.

### Live cell imaging, immunostaining, and SR-GSDIM

For live imaging, cells were exposed to 625 ng/mL Hoechst 33342 DNA stain (Sigma-Aldrich) for 20 min prior to imaging in growth medium containing phenol-red free FluroBrite DMEM (Invitrogen) and 20 mM 4-(2-hydroxyethyl)-1-piperazineethanesulfonic acid (HEPES). For immunostaining, cells were stained with primary antibody CTab (1:1,000) with or without anti-SATB1 (1:100) followed by AlexaFluor 647 (for 82-kDa ChAT) or AlexaFluor 542 (for SATB1) secondary antibodies. Cells were then counterstained with 2.5 μg/mL Hoechst.

For SR-GSDIM, cells were plated on 35 mm plates containing 22 × 22 mm 0.16–0.19 mm thick coverslips (VWR International, Mississauga, ON, Canada) prior to immunostaining. After staining, coverslips were placed face down on single depression glass slides (Gorilla Scientific, Gainesville, VA, USA) containing 100 mM of β-cysteamine (Sigma-Aldrich) and sealed with Twinsil^®^ silicon sealant (Picodent, Wipperfürth, Germany).

For confocal microscopy, digital images were acquired with a Zeiss LSM510-Meta laser-scanning confocal microscope (Carl Zeiss Canada Ltd., Toronto, ON, Canada) using a 63X oil-immersion objective (NA = 1.4) or an Olympus Fluoview 1000 (Olympus Canada Inc., Richmond Hill, ON, Canada) using a 60X oil-immersion objective (NA = 1.35). For live cell imaging, images were acquired using 405 nm excitation and 420–480 emission for Hoechst; and 488-nm excitation and 505-nm emission using a long-pass filter for 82-kDa ChAT-EGFP at 1024 × 1024 resolution. For immunostaining, images were acquired for Hoechst as above; 543-nm excitation and 560- to 615-nm band-pass emission filter for SATB1; and 647-nm excitation and 650-nm long-pass emission filter for 82-kDa ChAT. Images were processed in ImageJ^58^ where manipulations included filtering, thresholding, digital magnification, and deconvolution, then formatted in Adobe Illustrator (Adobe Systems Inc, San Jose, CA, USA).

For SR-GSDIM, digital images of fixed cells were acquired with a Leica super resolution ground-state depletion microscope (SR-GSD; Leica Microsystems Inc., Concord, ON, Canada) using a 160X oil-immersion objective, NA 1.47. Images were captured using the reversible saturable optical fluorescence transitions principle, as described^59^,^60^. Images were captured using the Leica Application Suite Advanced Fluorescence software, then processed on Imaris (version 7.5.0, Bitplane USA, Concord, MA, USA). Identification of 82-kDa ChAT clusters and quantification of cluster size and spacing was performed in Matlab (Mathworks, Natick, MA, USA) using a custom-written implementation of the Ordering Points To Identify Clusters algorithm^61^,^62^. Regular spacing of ChAT molecular clusters was detected by measuring the inter-cluster distance between each cluster and all other clusters in an image, and then performing a normalized Fast Fourier Transform to detect any regular cluster spacing.

To quantify co-localization on SR-GSDIM images, Imaris was used to create surface dots representing each protein, based on the estimated size for each protein and resolution of the image. We defined the protein size with the addition of both primary and secondary antibodies as 60 nm. Using the XT co-localize spots module, we set the threshold distance for co-localized dots to be 2/3rds the size of the smallest protein and computed the distance between each pair of dots, and thus the threshold for co-localization was set to 40 nm.

### Nuclear sub-fractionation and immunoblotting

Total, cytosolic, and nuclear subcellular fractions from cells treated with vehicle or Aβ_1–42_ were isolated as described previously^63^. Ten μg of protein from each of the whole cell extract, cytosolic (10 mM KCl) and soluble nuclear fractions, and half of the chromatin and insoluble protein fractions were separated on SDS–polyacrylamide gel electrophoresis (SDS-PAGE) gels.

For western immunoblotting experiments, total cell lysates were collected using a Triton-X lysis buffer (final concentration: 10 mM HEPES, 5 mM MgCl_2_, 1 mM ethylene glycol tetraacetic acid [EGTA], 150 mM NaCl, 5% [v/v] Glycerol, 0.5% [v/v] Triton-X 100, protease inhibitor cocktail [Sigma Aldrich; final concentration: 1.04 mM 4-(2-Aminoethyl) benzenesulfonyl fluoride hydrochloride (AEBSF), 0.8 μM aprotinin, 40 μM bestatin, 14 μM N-[N-(L-3-trans-carboxyirane-2-carbonyl)-L-leucyl]-agmatine; 1-[N-[(L-3-trans-carboxyoxirane-2-carbonyl)-L-leucyl]amino]-4-guanidinobutane (E-64), 20 μM leupeptin and 15 μM pepstatin], and phosphatase inhibitors [10 mM sodium fluoride, 1 mM sodium vanadate, 20 mM sodium phosphate, 3 mM β-glycerolphosphate, 5 mM sodium pyrophosphate]). Thirty μg of protein from each sample was separated on SDS-PAGE gels. The density of protein bands were determined using Image Lab (Version 5.0, BioRad), and compared to β-actin as a loading control.

### Chromatin immunoprecipitation with next-generation sequencing (ChIP-seq)

ChIP samples were obtained from 82-kDa ChAT and SATB1-expressing cells treated with vehicle or 100 nM Aβ_1–42_. ChIP was performed by cross-linking DNA and bound proteins with 0.5% methanol-free ultra-pure paraformaldehyde (Polysciences, Inc., Warrington, PA, USA). ChIP samples were analyzed at the Donnelly Sequencing Centre (Toronto, ON). ChIP-seq samples were individually barcoded with Illumina TruSeq^®^ adapter sequences (Illumina, San Diego, CA, USA) for library construction, then run on 2 single read 1:51bp lanes using a HiSeq2500.

ChIP-seq samples generated ten to twenty million 50 base pair, paired-end reads for each sample. Each ChIP-seq FASTQ file was loaded onto the Galaxy server (http://usegalaxy.org)^64^ for analysis. First, each sample was tested for quality control (QC) statistics, including quality scores, GC content and over-represented sequences. All the sequences were of sufficient quality, the GC content approximated the GC content of the human genome (~41%)^65^ and the only overrepresented sequences that were found corresponded to the adapter sequences used in the library construction and were at ~1% of the total reads. Reads were then mapped to the human genome (hg38, updated December 2013) using the Burrows-Wheeler Aligner^66^, with repeat-masking and default parameters. We also included a published^46^ human ATRX dataset (NCBI accession: GSE22162) that was mapped to hg19 using Bowtie2. Unique peaks were identified for each sample using the model-based analysis of ChIP-seq (MACS)^67^ tool with p-value < 0.00001, and filtered out significant peaks found in the corresponding input sample.

Peaks found by the MACS procedure were compared to genomic features obtained from the UCSC genome table browser^68^ using in-house SAS programs (version 9.4, Cary, NC, USA). SAS was used to compute average peak lengths for each sample and to annotate peaks found within a known gene. ChIP-seq tracks were visualized using the Intergrative Genomics Viewer (Broad Institute, Cambridge, MA, USA; version 2.3.59)^69^. These gene lists were uploaded to the DAVID server (http://david.abcc.ncifcrf.gov/)^37^,^38^ for GO analysis. Finally, FASTA formatted peaks were uploaded to the DREME tool (http://meme.nbcr.net/meme/cgi-bin/dreme.cgi)^28^ for motif discovery. SAS was also used in several downstream analyses to compute additional features of the ChIP-seq data. To visualize non-normal distribution of the distance between peaks, kernel density estimation was used, with a smoothing parameter of 0.5.

### Data Deposition

ChIP-seq data used in this study was deposited in the NCBI Geo Database, accession number: GSE73576.

### Quantitative PCR (qPCR)

For ChIP-qPCR, primers (Table S6) were incubated with DNA from either the primary antibody sample or corresponding input sample. Duplicate samples were incubated without primary antibody (IgG control). For reverse transcription-qPCR (RT-qPCR), total RNA was isolated from SH-SY5Y cells and reverse transcribed as described previously^20^. 1x iQ SYBR Green Supermix (Bio-Rad) was added prior to PCR amplification and real-time detection using a Bio-Rad C1000™ Thermal Cycler and CFX95 Real-Time system. After each cycle, fluorescent activity was determined, and a final crossing point (threshold cycle, Ct) was calculated. Primer efficiency and amplifications of a single PCR product was confirmed, and a representative gel for RT-PCR amplification can be found in Fig. S7. For ChIP-qPCR, the average Ct in each treatment and control was analyzed using the ΔΔCt method given by the formula: ΔΔCt (ChIP − IgG) = (Mean Ct (ChIP) − Mean Ct (Input)) − (Mean Ct (IgG) − Mean Ct (Input)). After determining the ΔΔCt, we used this to calculate a fold-change compared to the IgG control, given by the formula: Fold Change = 2^(−ΔΔCt (ChIP− IgG)). For RT-qPCR, the average Ct in each treatment was analyzed by the 2^^−ΔΔCt^ method, normalized to glyceraldehyde 3-phosphate dehydrogenase (GAPDH) and vehicle-treated samples.

### Data Analysis

The data are presented as mean ± SEM, with n values indicating the number of independent experiments performed with separate cell populations. Each n value represented the average of multiple sample replicates for each experiment. GraphPad Prism 5 (GraphPad Software, San Diego, CA, USA) was used for data analysis. Data were assessed for normality using Bartlett’s test and statistically significant differences were tested by unpaired Student’s *t*-test, or between groups using one or two-way ANOVA with Dunnett’s, Tukey’s or Bonferroni’s *post hoc* multiple comparison test as appropriate, with statistical significance defined as p ≤ 0.05. For cell counting, an independent and blinded observer determined the number of cells with nuclear aggregations on 63x magnification images. Each image had at least 20 cells, and 5 images were counted for each replicate of each treatment. The total number of cells in all 5 images was pooled to determine the percentage of cells with nuclear aggregations.

## Additional Information

**How to cite this article**: Winick-Ng, W. *et al*. 82-kDa choline acetyltransferase and SATB1 localize to β-amyloid induced matrix attachment regions. *Sci. Rep*. **6**, 23914; doi: 10.1038/srep23914 (2016).

## Supplementary Material

Supplementary Information

## Figures and Tables

**Table 1:** APP-interacting genes with 82-kDa ChAT and/or SATB1 association by ChIP-seq.

Gene	Function	82-kDa ChAT(Vehicle)	SATB1(Vehicle)	82-kDaChAT (Aβ)	SATB1(Aβ)	Microarray foldchange (ref. 23)
ADAM10	Alpha secretase proteolytic cleavage of APP	Intron 1	–	Intron 14	–	**2.4**
ADAM12	matrix metalloproteinase, multiple catalytic targets	Intron 3	–	Intron 2	–	n/a
ADAM17	MAP-kinase signalling, APP cleavage	Intron 1	–	–	–	**1.2**
APBA2 (x11β)	signal transduction, APP binding	–	Intron 3	Promoter	–	**2.2**
APBB1	APP binding, involved in APP signaling	Promoter	–	–	Intron 1	n/a
APBB2	APP binding, involved in signaling	Intron 1	–	Intron 6	Intron 10	n/a
APP	β-amyloid precursor, synapse maintenance	–	–	Intron 13	Intron 13	n/a
APPBP2	Binds to APP intracellular domain	Intron 4	–	–	–	**1.4**
NAE1	APP binding, can drive APP-mediated apoptosis	–	–	Intron 7	–	n/a
RTN1	Modulates β-secretase activity, APP binding near cleavage site	–	Intron 1	Intron 1, 3	–	**14.8**
