# Supplementary material for: 82-kDa choline acetyltransferase and SATB1 localize to β-amyloid induced matrix attachment regions
Source: Sci Rep. 2016 Apr 7;6:23914. doi: 10.1038/srep23914 (PMC4823725; doi:10.1038/srep23914)
Supplement: Supplementary Information [file srep23914-s1.pdf]

82-kDa choline acetyltransferase and SATB1 localize to β-amyloid induced matrix attachment regions

Warren Winick-Ng, Fabiana A Caetano, Jennifer Winick-Ng, Trevor M Morey, Bryan Heit and R. Jane Rylett

Supplemental Figures and Tables

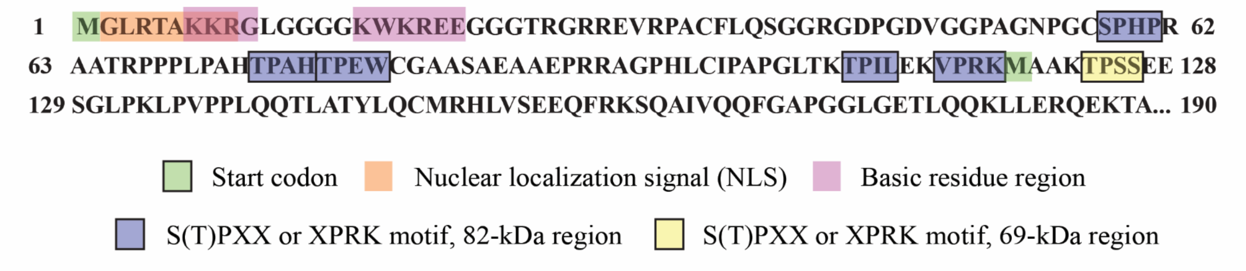

Supplementary Figure S1. **DNA binding prediction for 82-kDa ChAT.**

The 118 amino acid residue amino-terminus of 82-kDa ChAT is flanked by two in-frame methionine initiation sites, and contains a unique nuclear localization sequence. Several DNA binding prediction software databases predicted high DNA binding probability at 2 basic residue regions, as well as 5 SPXX/XPRK DNA binding motifs. There was an additional SPXX motif in the 69-kDa ChAT region near the amino terminus with high DNA binding prediction.

A

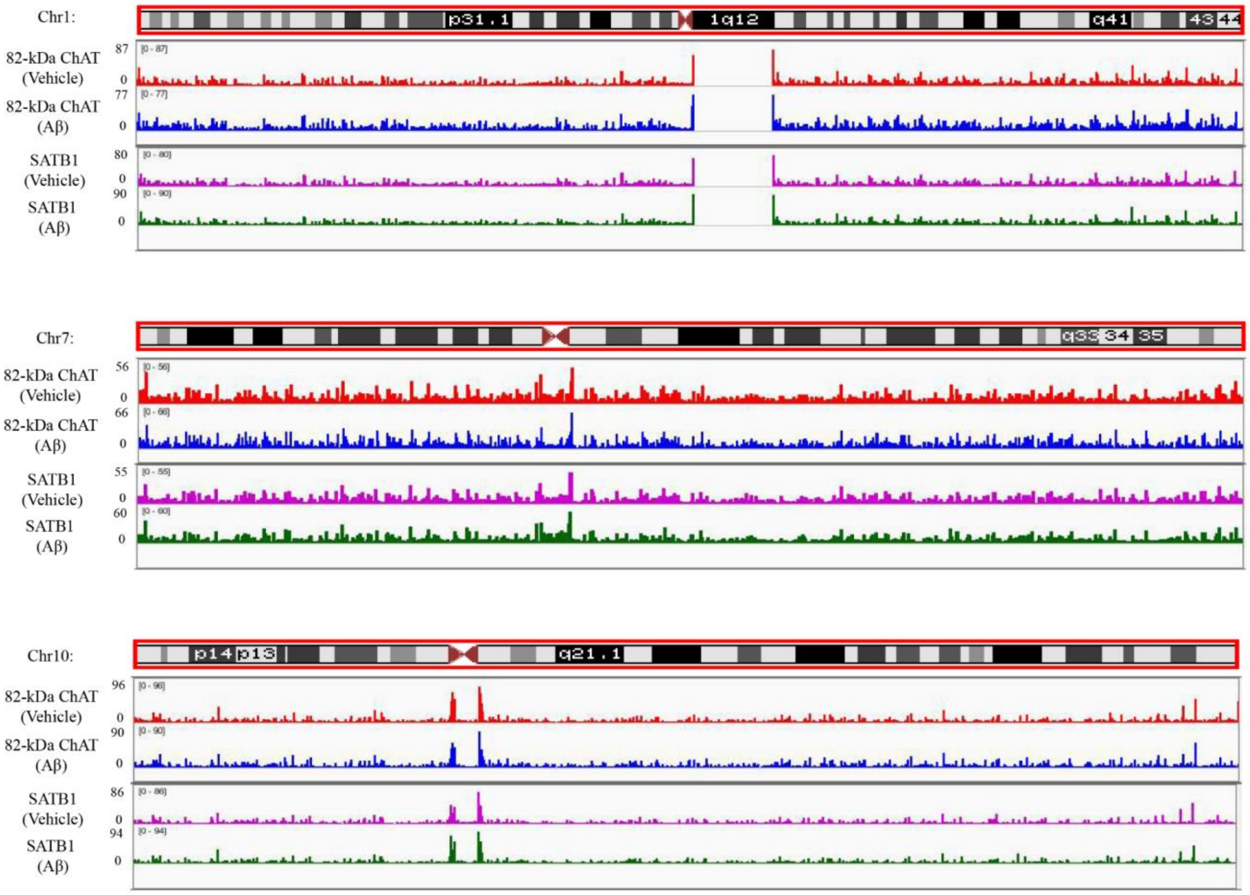
Chr1:
82-kDa ChAT (Vehicle)
82-kDa ChAT (Aβ)
SATB1 (Vehicle)
SATB1 (Aβ)
Chr7:
82-kDa ChAT (Vehicle)
82-kDa ChAT (Aβ)
SATB1 (Vehicle)
SATB1 (Aβ)
Chr10:
82-kDa ChAT (Vehicle)
82-kDa ChAT (Aβ)
SATB1 (Vehicle)
SATB1 (Aβ)

B

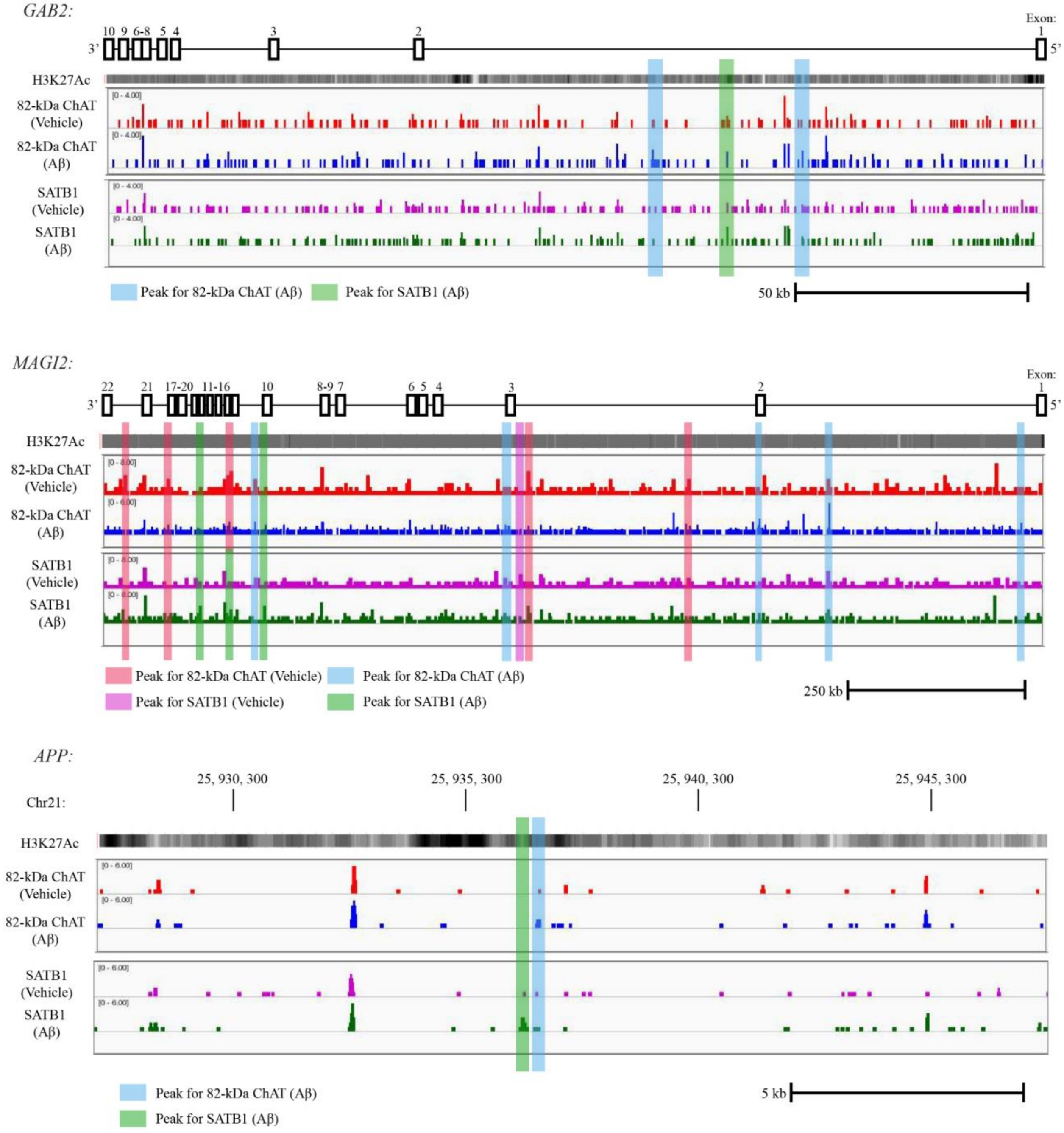

Supplementary Figure S2. **Examples of ChIP-seq tracks for 82-kDa ChAT and SATB1**.

(A) ChIP-seq tracks for chromosomes 1, 7 and 10 for 82-kDa ChAT and SATB1 in cells treated with either vehicle or $A\beta_{1-42}$. For chromosome 1, there was a portion of data not covered at the

start of the q-arm. No other chromosome had a gap in data coverage. For chromosomes 7 and 10, there was a large peak of coverage flanking the centromeres. (B) ChIP-seq tracks for *GAB2* and *MAGI2* whole genes, as well as for a region of *APP*, for 82-kDa ChAT and SATB1 in cells treated with either vehicle or $A\beta_{1-42}$. Peaks are highlighted in blue for 82-kDa ChAT $A\beta_{1-42}$ peaks, green for SATB1 $A\beta_{1-42}$ peaks, red for 82-kDa ChAT vehicle peaks, and purple for SATB1 vehicle peaks. H3K27Ac is overlayed to show active transcription initiation sites

Supplementary Table S3. Motifs used in S/MAR analysis. Motifs 1-18 were used previously to determine S/MAR association (47), motifs 19-21 are SATB1 binding motifs (36) and motifs 22-26 were identified in a D. melanogaster S/MAR study (48).

Number	Name	Motif
1	Origin of Replication Signal 1	*ATTA*
2	Origin of Replication Signal 2	*ATTTA*
3	Origin of Replication Signal 3	*ATTTTA*
4	TG-Rich Signal 1	*TGTTTTG*
5	TG-Rich Signal 2	*TGTTTTTTG*
6	TG-Rich Signal 3	*TTTTGGGG*
7	Curved DNA Signal 1	$AAAA(N_7)AAAA(N_7)AAAA$
8	Curved DNA Signal 2	$TTTT(N_7)TTTT(N_7)TTTT$
9	Curved DNA Signal 3	*TTTAAA*
10	Kinked DNA Signal 1	$TA(N_3)TG(N_3)CA$
11	Kinked DNA Signal 2	$TA(N_3)CA(N_3)TG$
12	Kinked DNA Signal 3	$TG(N_3)TA(N_3)CA$
13	Kinked DNA Signal 4	$TG(N_3)CA(N_3)TA$
14	Kinked DNA Signal 5	$CA(N_3)TA(N_3)TG$
15	Kinked DNA Signal 6	$CA(N_3)TG(N_3)TA$
16	mtopo-II Signal	*(A/G)N(T/C)NNCNNG(T/C)NG(G/T)TN(T/C)n(T/C)*
17	dtopo-II Signal	*GTN(A/T)A(T/C)ATTNATNN(A/G)*
18	AT-Rich Signal	$(A/T)_6$
19	SATB1 Motif 1	$(A/T)_{3\text{-}n}(C/G)(A/T)_{3\text{-}6}$
20	SATB1 Motif 2	$(A/T/[C\ OR\ G])_{20+}$
21	SATB1 Motif 3	$(A/T)_{2+}(C)(A/T)_{2+}$; *palindrome*
22	Drosophila MAR Motif 1	A_{12+}
23	Drosophila MAR Motif 2	$(AG)_8$ *or* $(AC)_8$
24	Drosophila MAR Motif 3	$(AGC)_5$
25	Drosophila MAR Motif 4	$(AACAGC)_2$
26	Drosophila MAR Motif 5	$(AAAAA[C/G/T])_2$

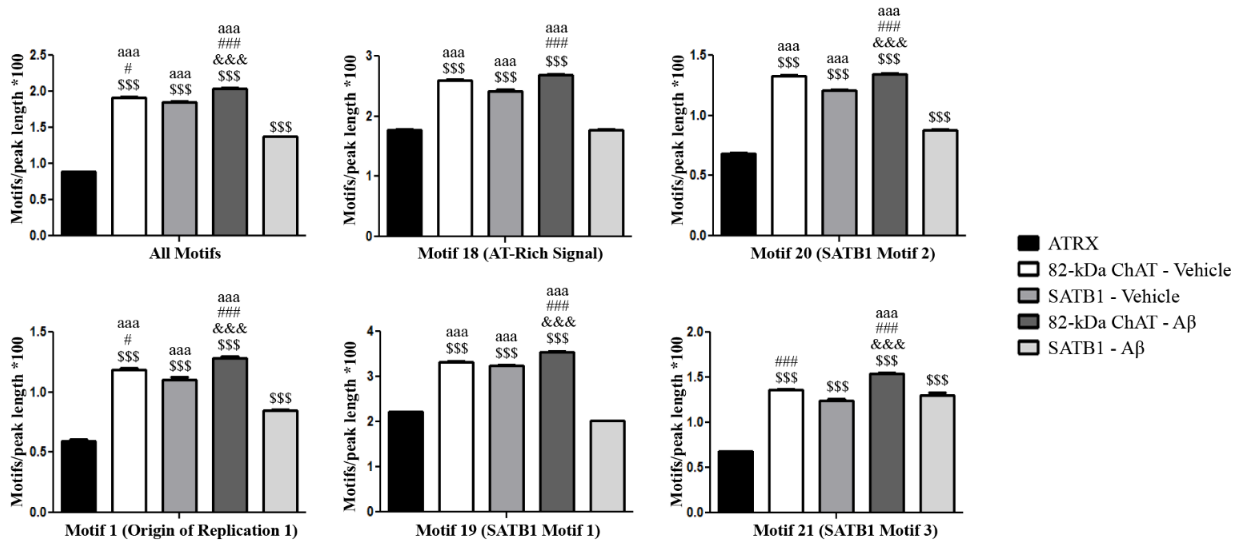

Supplementary Figure S4. **82-kDa ChAT and SATB1 associate with chromatin at S/MARs.** Mean weighted S/MAR motifs (**Table S3**)/ChIP-seq peak computed as the number of motifs/peak length*100. Both vehicle and Aβ_{1-42}-treated cells for 82-kDa ChAT and SATB1 had a higher number of weighted motifs/peak compared to ATRX for all motifs. A similar pattern was found for the 5 highest represented motifs. $$$ $p < 0.001$ compared to ATRX, aaa $p < 0.001$ compared to Aβ_{1-42}-treated SATB1, &&& $p < 0.001$ compared to vehicle-treated 82-kDa ChAT, and ### $p < 0.001$ and # $p < 0.05$ compared to vehicle-treated SATB1.

Supplementary Table S5. G-quadraplex ($G_{3+}N_{1-20}G_{3+}N_{1-20}G_{3+}N_{1-20}G_{3+}$) motifs found in ChIP-seq datasets.

Sample	Peaks with G-Quadraplex motifs	Total G-Quadraplex motifs
ATRX	732	1327
82-kDa ChAT (Vehicle)	113	204
SATB1 (Vehicle)	60	109
82-kDa ChAT (Aβ)	89	121
SATB1 (Aβ)	30	41

Supplementary Table S6. ChIP-qPCR and RT-qPCR primers.

Gene	Direction	Primer, 5'-3'
ChIP-qPCR		
Human APP	Forward	GCCAGACCACAACCTCGTTT
	Reverse	TTGGGAACTTGCGTGGCAAA
RT-qPCR		
Human SATB1	Forward	GTAGAGCTAGCGAGGGAGAGA
	Reverse	TTGTTGTTGTGACGAGGCCG
Human Total APP	Forward	AACCAGTGACCATCCAGAAC
	Reverse	ACTTGTCAGGAACGAGAAGG
Human APP-KPI	Forward	GTCTGTGGAAGAGGTGGTTC
	Reverse	GTCAAAGTTGTTCCGGTTG
Human GAPDH	Forward	TGTTGCCATCAATGACCCCTT
	Reverse	CTCCACGACGTACTCAGCG

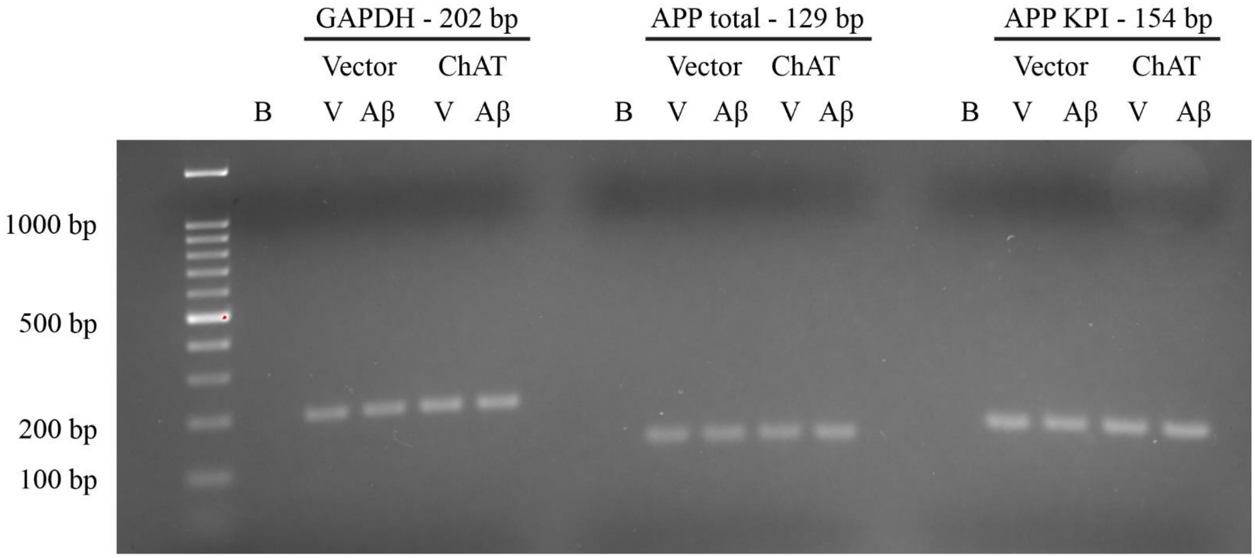

Supplementary Figure S7. **Representative PCR gel for primers used in RT-qPCR experiments.** SH-SY5Y cells stably expressing either an empty vector or 82-kDa ChAT were treated with either vehicle or 100 nM oligomeric $A\beta_{1\text{-}42}$. Total RNA was extracted and reverse transcribed prior to PCR amplification with primers specific to *GAPDH*, total *APP* or *APP-KPI* mRNA. For all primer sets, there was a single PCR product at the predicted size.
